# A Custom Hinged Endoprosthesis for the Treatment of Proximal Tibial Osteosarcoma in Skeletally Immature Patients

**DOI:** 10.3390/cancers17121952

**Published:** 2025-06-12

**Authors:** Zhiqing Zhao, Qi Han, Jichuan Wang, Wei Wang, Wei Guo, Taiqiang Yan

**Affiliations:** 1 Department of Orthopedics, Peking University First Hospital, No. 8 Xishiku Street, Xicheng District, Beijing 100034, China; zhaozhiqing@pku.org.cn (Z.Z.); 20929@pkufh.com (Q.H.); 2011110359@bjmu.edu.cn (W.W.); 2 Musculoskeletal Tumor Centre, Peking University People’s Hospital, No. 11 Xizhimen South Road, Xicheng District, Beijing 100044, China

**Keywords:** children, osteosarcoma, proximal tibia, endoprosthesis, limb salvage, limb length discrepancy

## Abstract

**Background:** The optimal treatment for proximal tibial osteosarcoma (OS) in skeletally immature patients remains controversial. A custom hinged endoprosthesis has been used to preserve the growth potential of the distal femur. This study aims to report (1) the 2-year follow-up outcomes after surgery for pediatric proximal tibial OS; (2) the complications associated with this endoprosthesis; and (3) the extent to which the growth potential of the adjacent open physis can be preserved. **Methods:** Seven skeletally immature patients (mean age, 11.1 years; range, 9–13 years) with proximal tibial OS were included between November 2020 and December 2022. All underwent tumor resection and reconstruction by this custom endoprosthesis. Postoperative limb function was evaluated by the Musculoskeletal Tumor Society (MSTS) score system and complications were recorded. Overall leg length and femoral length were measured radiographically to determine the growth rate. **Results:** The mean follow-up time was 34.7 months (standard deviation (SD), 8.9 months). One patient presented with local recurrence 12 months after surgery, and another patient had pulmonary metastasis 3 months postoperatively. The range of flexion of the knee after rehabilitation was between 90° and 125°, with an average of 103.6° (SD, 12.5°). The average MSTS score of the patients after surgery was 27.4 (SD, 1.5). Wound dehiscence took place in three patients after chemotherapy. At the last follow-up, the overall limb length discrepancy was 2.1 cm (SD, 2.4 cm). Growth at the distal femoral physis after surgery was observed in all patients during follow-up, with an average of 81.4% (range, 57.78–100%) of growth of the contralateral distal femoral physis. **Conclusions:** This custom hinged endoprosthesis can preserve the growth potential of the adjacent distal femur and provide satisfying functional outcomes with lower postoperative complication rate. It could serve as an alternative for proximal tibial OS in skeletally immature children.

## Simple Summary

Proximal tibial reconstruction is one of the most difficult procedures in limb-preserving surgery for pediatric patients. The resection of the physes in skeletally immature children often results in limb length discrepancy. A custom hinged endoprosthesis was designed for pediatric limb salvage with the preservation of growth originating from the ipsilateral distal femur. The endoprosthesis is intended to be temporary and is eventually converted to an adult modular oncological prosthesis once the child has reached skeletal maturity. This study reported the outcomes of patients treated with this endoprosthesis. The results demonstrate that this custom hinged endoprosthesis can preserve the growth plates in the adjacent femur and provide satisfying functional outcomes with lower postoperative complication rate. It could serve as an alternative for proximal tibial osteosarcoma in skeletally immature children.

## 1. Introduction

Osteosarcoma (OS) is the most common primary malignant bone tumor in pediatrics. Usually, it occurs in the metaphyseal region of long bones, with more than 50% of the cases occurring around the knee [1]. For skeletally immature patients with OS, amputation has in the past been considered the main option [2,3]. However, approximately 85 percent of children have received limb salvage surgery due to the rising survival rate following multiagent chemotherapy and surgical techniques [4,5,6,7,8,9]. Proximal tibial reconstruction is one of the most difficult procedures in limb-preserving surgery for pediatric patients. None of the available options, whether biological or non-biological, are optimal. Therefore, controversy remains regarding this issue. The resection of the physes in skeletally immature children will result in limb length discrepancy (LLD). Consequently, functional deficit, gait disturbances, secondary pelvis obliquity, and cosmetic issues occur.

To combat the problem of LLD, different models of expandable prosthesis have been designed, as prior studies have reported, including invasive prosthesis and the recently introduced non-invasive expandable prosthesis [8,10,11,12,13,14,15,16,17]. However, the high complication rate, including loosening, neuropraxia, and notably infection, is not comparable with that associated with a static prosthesis. An average of 3.2 complications per patient has been documented for extendible endoprostheses [18]. In addition to the high complication rate of extendible prostheses, 8% to 50% of patients do not undergo a lengthening procedure due to tumor progress [19,20,21,22]. The total cost of expandable endoprosthesis is considerable, and has been reported to be as high as USD 379,000 [23]. Also, in some situations, an expandable prosthesis is not available and is therefore not an option. Considering the higher complication rate and the high cost of extendible endoprostheses, some researchers advocate temporary hemiarthroplasty and staged lengthening, with advantages including the simple technique and the low cost [24,25].

Mostly, for OS located in the proximal tibia, the growth plate of the proximal tibia should be removed to ensure the complete resection of the tumor, except where the tumor margin is 1 cm away from the proximal growth plate. At the authors’ institution, a custom hinged endoprosthesis was used for pediatric limb salvage with the preservation of growth originating from the ipsilateral distal femur. The endoprosthesis is intended to be temporary and is eventually converted to an adult modular oncological prosthesis once the child has reached skeletal maturity. We report on a relatively homogeneous group of patients, treated with this endoprosthesis. A minimum follow-up of 2 years was required for inclusion, unless death from tumor progression occurred earlier. In this study we aimed to show (1) the clinical results after reconstruction by the custom endoprosthesis; (2) the complications associated with this endoprosthesis; and (3) the extent of growth potential that can be preserved in the adjacent open physis.

## 2. Methods

### 2.1. The Description of the Endoprosthesis

**Figure 1:**
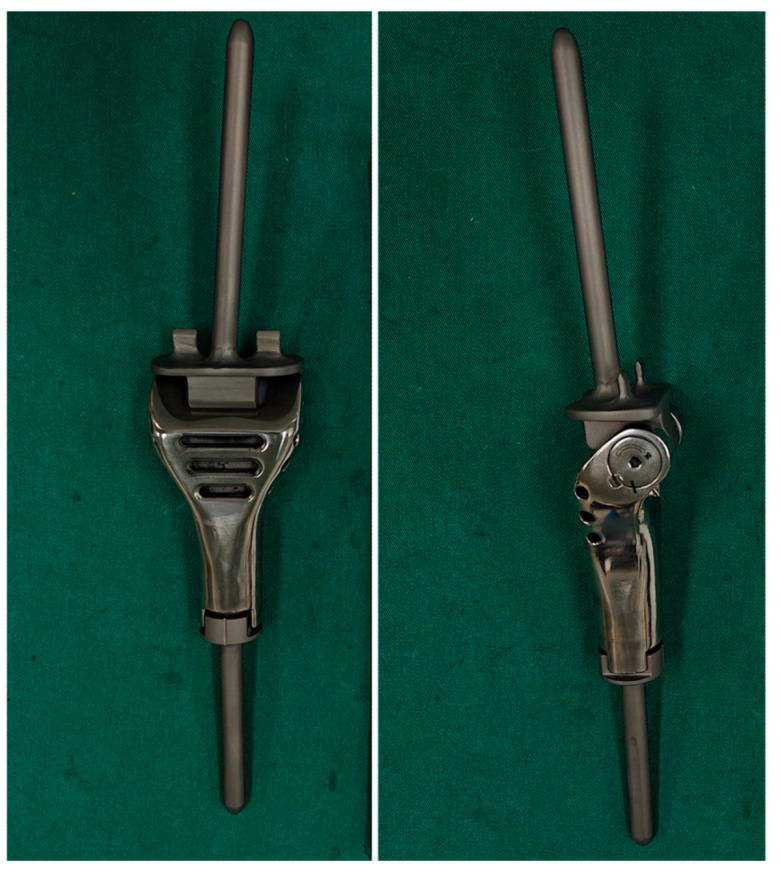
The intraoperative photos show the special hinged-joint prosthesis.

The endoprosthesis (produced by the *Lidakang* corporation, Beijing, China) consists of a tibial component with a fixed length of segmental defect body made of a cobalt–chromium alloy. It also includes a distal femoral component made of the Ti-6Al-4V titanium alloy. The femoral component is designed as a hinged base plate and a small-diameter (8, 9, or 10 mm) polished stem with a 5° valgus. The length of the stem is 100 mm. The anchorage of the femoral component necessitates the penetration of the adjacent femur through the growth plate. This is achieved by using an intramedullary stem with a smooth, polished surface that spans the unresected physis and is implanted without the use of cement. Furthermore, there are two anti-rotation needles on both sides of the femoral plateau, each measuring 10 mm in width and 12 mm in length. The anti-rotation needles can penetrate the epiphysis of the distal femur without crossing the growth plate (Figure 1).

### 2.2. Patients

Between 2020 and 2022, 10 children with OS underwent the procedure using the above-mentioned prosthesis. Skeletal immaturity was defined as follows: (i) age younger than 14 years; (ii) the presence of an open growth plate; and (iii) expected LLD >3 cm, based on the curve determined by Anderson et al. [26]. This study was approved by the ethics review committee of the institution (No. 2023PHB413-001, approval date: 15 March 2024.), and informed consent was obtained from the patients’ guardians. All surgical procedures were performed by the same team of trained orthopedic oncology surgeons (YTQ and ZZQ). Two patients with incomplete data were excluded, and one patient who died of pulmonary metastasis 12 months after surgery was also excluded. Of the remaining seven patients, three were boys and four girls, with an average age of 11.1years (standard deviation (SD), 1.5 years; range, 9–13 years) at the time of diagnosis (Table 1).

### 2.3. Treatment Strategy

Diagnosis of OS was confirmed by percutaneous bone biopsy for all cases. Standard treatment was administered to all patients, including neoadjuvant chemotherapy, surgery, and postoperative chemotherapy. The surgery was performed using an extensile lateral parapatellar approach. An average of 12.3 cm (SD, 3.7 cm; range, 8–16 cm) of proximal tibia was resected.

**Figure 2:**
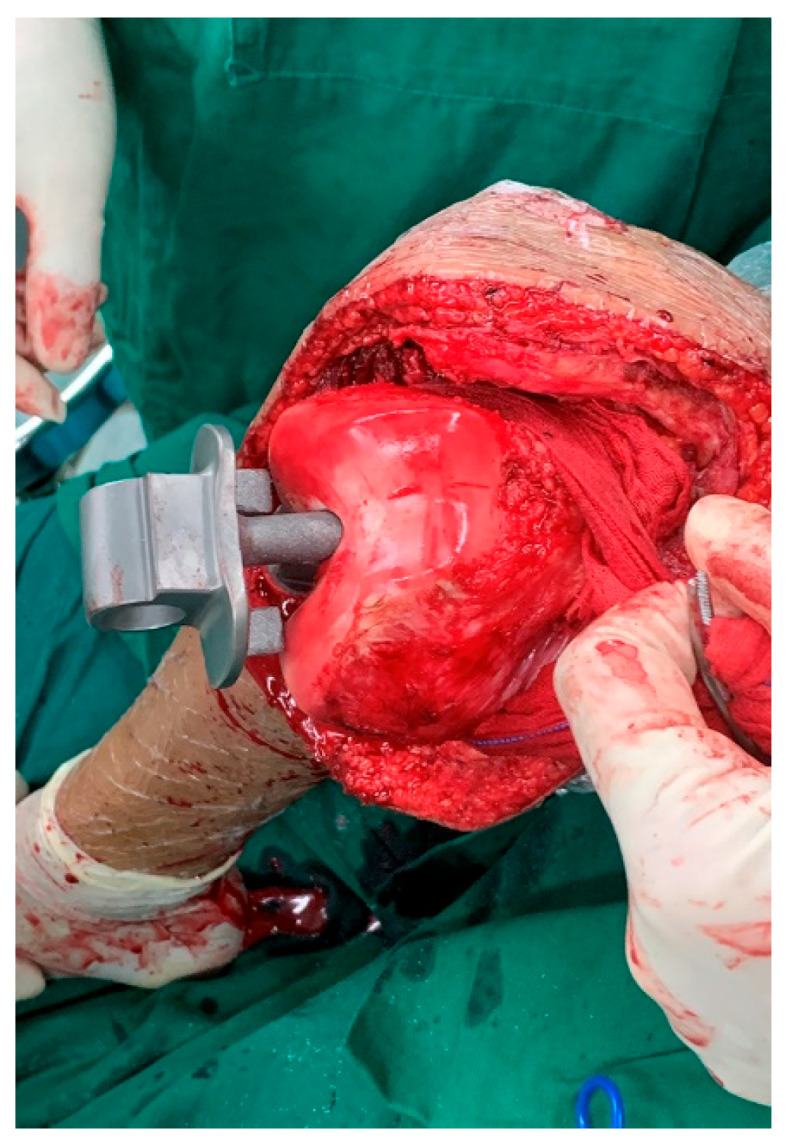
The condylar cartilage of the femoral condyle was retained, and the stalk penetrates the femoral condyle and is directly inserted into the medullary cavity without bone cement.

During the preparation of the distal femur, we advertently left behind the condylar cartilage of the distal femoral condyle. We proceeded to open the femoral canal using a 6 mm awl, followed by minimal reaming and the fixation of the femur component. The prosthesis stalk has a smooth surface and does not remove bone during reaming. After determining the internal and external rotation angles, the femoral stalk penetrates the femoral condyle and is directly inserted into the medullary cavity without bone cement (Figure 2).

**Figure 3:**
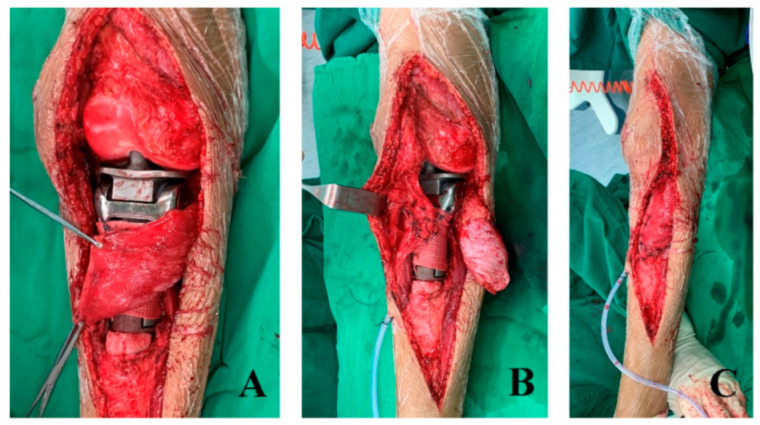
The photos show that gastrocnemius rotation flap reconstruction was performed (**A**); the extensor mechanism was reconstructed using an artificial ligament (**B**); and the gastrocnemius rotation flap was wrapped around the endoprosthesis (**C**). (Patient No. 1).

In all patients, reconstruction was performed allowing 1 cm or 2 cm longer than the actual defect to compensate for contralateral growth potential. The extensor mechanism was repaired by the suturing of the patellar tendon to the hole in the tibial prosthesis and wrapping the artificial ligament around the prosthesis (Figure 3). Gastrocnemius flap reconstruction was undertaken to cover the endoprosthesis in all cases.

Postoperatively, patients began ambulation around one week after the surgery, but the knee should be kept straight with a brace. Knee flexion exercises began 6 weeks after surgery.

### 2.4. Data Collection

We recorded in detail the clinical data of the following three aspects: (1) preoperative information, including demographic information such as the patient’s age, gender, and diagnosis; (2) intraoperative data, including surgical parameters such as osteotomy length and total prosthesis length; and (3) postoperative data covering indicators such as oncology prognosis, functional recovery, complications, overall LLD, and femoral length. The follow-up period was calculated from the day of the operation and ended at the last follow-up or the patient’s death or amputation (for limb survival analysis).

All measurement data were completed by the same researcher (WJC) using electronic measurement methods after calibration in accordance with the implant specification document. The functional assessment was conducted using the Musculoskeletal Tumor Society (MSTS) scoring system [27]. This scale comprehensively evaluates six dimensions, including the degree of pain, functional status, the psychological acceptance of functional deficits, walking ability, gait characteristics, and the use of walking aids.

The length data were collected by clinical examination and by the measurement of radiographs with a magnification scale, which enables accurate measurements of the true distances. Femoral growth was assessed by full-length standing anteroposterior radiographs of bilateral lower limbs with the patella pointing anteriorly (teleoroentgenogram) [28], which utilize three radiographic exposures centered on the hip, knee, and ankle joints. With this technique, the patient remains supine, with both patellae pointing toward the ceiling [29]. Femoral length and overall leg length were measured by one of the authors (HQ), with the final femur and LLD verified by the senior author (GW). Overall leg length was measured from the top of the femoral head to the center of the tibial plafond. The femoral length of the operative leg was measured from the center of the medial prosthetic plateau to the top of the femoral head. The femoral length of the contralateral limb was measured from the central position of the femoral condyle to the top of the femoral head. The LLD is the difference in the length of one leg compared to the other.

## 3. Results

### 3.1. Oncological Outcome

No patients were lost to follow-up and the average follow-up duration was 34.7 months (2.9 years) (range, 24–47 months). One patient presented with local recurrence 12 months after surgery, and she received above-knee amputation. The endoprosthesis was functioning well at that time. One patient had pulmonary metastasis 3 months after surgery and received chemotherapy, but is still alive with the disease (Table 2).

### 3.2. Functional Outcome

**Figure 4:**
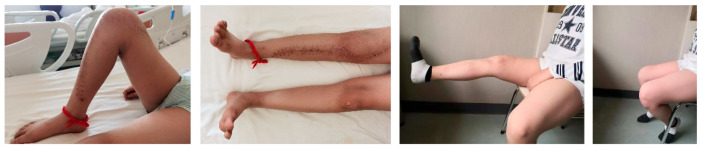
The range of motion of the knee after rehabilitation was satisfied (patient No. 2).

The alive children can walk stably without pain, go back to school, return to their entertainment activities, and even engage in running or biking. The range of flexion of the knee after rehabilitation was between 90° and 125°, with an average of 103.6° ± 12.5° (Figure 4). The average MSTS score of the alive patients was 27.4 (SD, 1.5; range, 25–29). No knee instability was found during the last follow-up.

### 3.3. Complications

**Figure 5:**
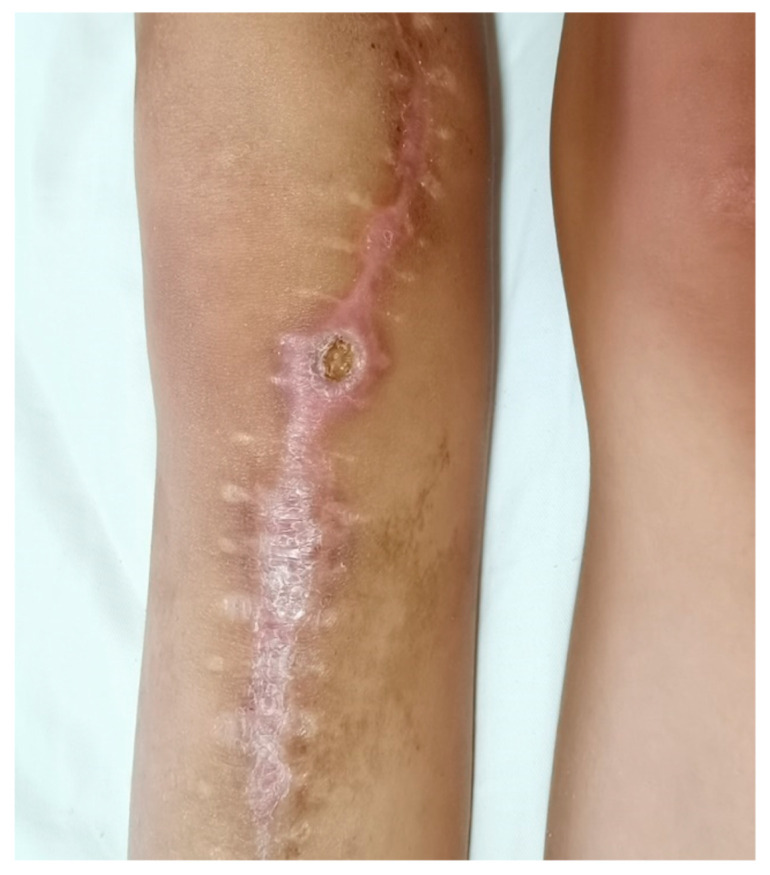
The photo shows wound dehiscence after surgery.

Overall, three patients suffered from wound dehiscence (Figure 5). The wound dehiscence was solved by dressing the wound and antibiotics in two cases, while another was successfully treated by debridement and antibiotics. One patient presented with local relapse after surgery. Thereafter, amputation was performed and she was alive without disease.

### 3.4. Femoral Discrepancy

**Figure 6:**
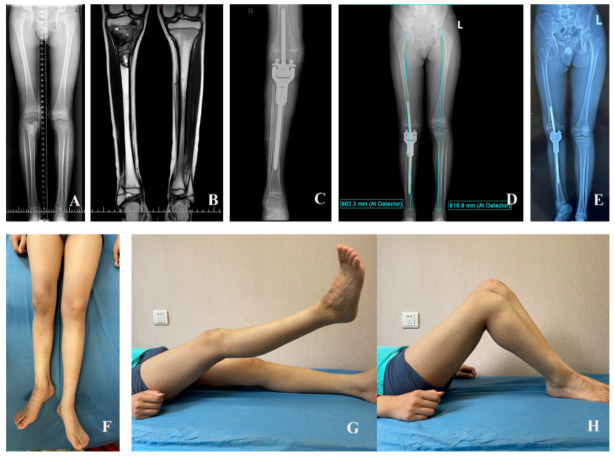
The preoperative X-ray film shows a 10-year-old boy (patient No. 1) with osteosarcoma of the right proximal tibia (**A**); postoperative MRI (**B**); postoperative X-ray film (**C**); the full-length standing anteroposterior radiographs were taken 12 months (**D**) and 45 months (**E**) after the initial surgery; the range of motion of the knee after rehabilitation was satisfied (**F**–**H**).

At the last follow-up, the overall LLD was 2.1 cm (SD, 2.4 cm; range, −0.6–6 cm). Growth at the distal femoral physis after surgery was observed in all of the patients during follow-up, with an average of 81.4% (SD, 15%; range, 57.78–100%) of growth of the contralateral distal femoral physis. Five patients exhibited femoral length shortening. The mean reduction in femur length was 1.0 cm (SD, 1.0 cm; range, 0–2.7 cm) (Figure 6).

## 4. Discussion

For proximal tibial OS, there are many available treatment strategies, but the optimal reconstruction choice in children at this site remains controversial. Rotationplasty results in excellent functional success in children [30]; however, the acceptability of patients or their guardians was very low due to psychological and social factors [31]. Excellent stability can be achieved by arthrodesis, but it has the weakness of a lack of knee motion [32]. Reconstruction by allograft exhibits some complications, including deep infection, allograft fracture, non-union, and the instability of the joint [33,34,35]. For skeletally immature children, the sacrifice of major growth plates during tumor resection causes significant LLD as growth progresses. Expandable endoprostheses have been widely reported on in previous studies [16,21,36]; however, they are always associated with high complication rates. Moreover, up to 50% of patients treated with expandable endoprosthesis do not receive subsequent lengthening procedures due to oncological failures or the overestimation of expected LLD [24,37,38,39,40]. In our center, to address LLD, a custom hinged endoprosthesis has been introduced with the aim of saving the growth ability of the distal femur. Our findings indicate that the distal femoral physis still has growth potential, partially or normally, after the polished stem is inserted through the physis.

The new prosthetic replacement represents a significant advancement in treatment over time. At the authors’ institution, hemiarthroplasty has been utilized in order to protect the growth plate of the unaffected distal femur in recent decades. Due to the loss of the main ligaments of the knee, stability was jeopardized and compensated for by scar tissue and high adaptability in children [9]. To improve the stability of hemiarthroplasty in limb salvage for skeletally immature patients, this new hinged endoprosthesis was introduced. The femoral component of the prosthesis has a polished stem which may save the potential of femoral growth. Actually, it was inspired by most extendable tumor prostheses for children having a polished, press-fit stem passing through the central portion of the uninvolved adjacent physis [21,22,36,41,42]. Neel et al. reviewed six patients with OS and found that the polished stem will not result in growth retardation or arrest [43]. A previous study conducted by our team indicated that the proximal tibial physis showed an average of 74.3% (range, 30–100%) of growth potential compared with the unaffected proximal tibial physis after using a small-diameter polished stem (*n* = 34) [42]. In the current study, an average growth potential preservation of 83.7% was observed in the distal femoral physis. Three patients (37.5%) had exactly the same length of longitudinal growth in the salvaged limb compared to the unaffected one. Our findings show that the growth ability could be retained with the use of a polished stem. However, the majority of cases may not acquire a normal growth ability after the implantation of a femoral stem. The reasons for why the degree of femoral growth varied are unclear. Further study is needed to explain this.

In the current study, postoperative limb function was satisfied in all patients, with an average MSTS score of 27.4 (91.3%), which is comparable to previous published series treated with a polished tibial stem. Several clinical studies have reported favorable functional outcomes following expandable prosthesis reconstruction. A study conducted by Zou et al. [40] reported the outcomes of custom-made extendible endoprostheses in 55 children with sarcoma around the knee. A mean total lengthening of 4.2 cm was achieved with an excellent mean functional score of 83.2% [44]. In a multicenter study by Neel et al. [16], patients achieved excellent postoperative function with a mean MSTS score of 90% using non-invasive expandable prostheses. Similarly, Ji et al. [45] demonstrated good functional results in skeletally immature patients, reporting a mean MSTS score of 80.6% (range, 60–90%) in their series of 12 cases reconstructed with small-diameter polished tibial stem non-hinged endoprostheses. Compared to expandable endoprostheses, this custom hinged endoprosthesis was economical in the domestic market, costing only USD 8700. Furthermore, this new hinged endoprosthesis provides better stability than non-hinged ones. Therefore, some patients in our study can engage in sports activities, such as running, biking, or playing badminton.

The literature documented significant revision rates (22–83%) for invasive expandable endoprostheses, primarily due to aseptic loosening, prosthetic fracture, or deep infection [16,20,21,22,46,47,48]. While non-invasive expandable designs have recently demonstrated certain advantages over their invasive counterparts, they still maintain concerningly high complication rates [36,49]. Wilkins et al. [25] specifically highlighted the inherent mechanical limitations of expandable endoprostheses when compared to static prostheses. Additionally, the progressive bone loss around the stem of expandable implants frequently compromises future revision options, often necessitating conversion to total femoral replacement [50]. In contrast to these reported outcomes, our current series demonstrated superior performance, with no cases requiring revision surgery for infection, loosening, or prosthetic fracture. This complication profile appears more favorable than that associated with expandable endoprostheses. Several factors may explain the clinical preference for custom hinged endoprostheses over expandable designs. Most importantly, the static nature of hinged prostheses eliminates the need for repeated surgical interventions required by expandable systems. Additionally, the endoprosthesis preserves a greater amount of bone stock for future revision surgery compared to the expandable one. Moreover, the follow-up time is relatively short.

Several limitations should be considered when interpreting our study findings. First, the retrospective design and relatively small sample size may limit the generalizability of our results. Second, the mean follow-up period remains relatively short for evaluating long-term outcomes, which is attributable to the fact that this newly designed hinged endoprosthesis only became commercially available in 2020. Additionally, our study exclusively focused on pediatric patients with proximal tibial OS, potentially restricting the broader applicability of our conclusions. Third, the reliance on radiographs for tibial length measurements introduces potential measurement inaccuracies, particularly when knee flexion leads to foreshortening effects that may underestimate true tibial length. While we instructed patients to maintain a supine position with full knee extension during imaging, this methodological limitation persists.

## 5. Conclusions

This custom hinged endoprosthesis can preserve the growth potential of the adjacent femur, provide satisfying functional outcomes with lower postoperative complication rate. It could serve as an alternative for proximal tibial OS in skeletally immature patients. However, for cases where the tumor margin is more than 1 cm from the epiphysis, joint-preserving reconstruction may still be considered.

## Figures and Tables

**Table 1:** Patients’ demographics and surgical information.

No.	Age/Sex	Side	Enneking Stage	Operation Time (min)	Blood Loss (mL)
1	10/M	Right	IIB	155	150
2	10/M	Right	IIB	180	200
3	9/F	Right	IIB	190	300
4	13/F	Right	IIB	160	200
5	12/F	Right	IIB	190	400
6	12/F	Right	IIB	170	200
7	12/M	Right	III	175	450

M: male; F: female.

**Table 2:** Surgical data and postoperative outcomes.

No.	Resection Length (cm)	Reconstruction Length (cm)	Total LLD (cm)	Longitudinal Growth of Femur (cm)		Complication	MSTS	Follow-Up Time (Month)	End
				Salvaged (% of NO)	Non-Operated				
1	8	9	6	2.6 (57.78)	4.5		29	47	NED
2	16	17	3.3	3.8 (82.61)	4.6		29	43	NED
3	8	10	4.2	4.7 (73.44)	6.4	Wound dehiscence (7 months)	27	41	NED
4	16	17	1	2 (80)	2.5		26	32	AWD
5	12	13	0	6.2 (100)	6.2	Wound dehiscence (6 months)	28	29	NED
6	16	17	−0.6	1.2 (100)	1.2		28	27	NED
7	10	10	1.1	2.5 (75.76)	3.3	Wound dehiscence (1 month)	25	24	AWD

NED: no evidence of disease; AWD: alive with disease.

## Data Availability

The datasets used and/or analyzed during the current study are available from the corresponding author on reasonable request.
